# Investigating the Efficacy of Telerehabilitation in Individuals Who Underwent Hand Tendon Surgery: A Randomized Controlled Trial

**DOI:** 10.63144/ijt.2026.6752

**Published:** 2026-06-01

**Authors:** Zerrin İzmirli Mete, Ferruh Taşpınar, Yasemin Özkan, Betül Taşpınar

**Affiliations:** 1 Department of Physiotherapy and Rehabilitation, Institute of Health Sciences, Izmir Democracy University, Izmir, Türkiye; 2 Department of Physiotherapy and Rehabilitation, Faculty of Health Sciences, Izmir Democracy University, Izmir, Türkiye; 3 Department of Physical Therapy and Rehabilitation, Faculty of Medicine, Aydın Adnan Menderes University, Aydın, Türkiye

**Keywords:** Exercise, Injury, Telerehabilitation, Tendon, Treatment

## Abstract

Hand tendon injuries require a long rehabilitation process. Telerehabilitation may offer advantages in terms of accessibility, time, and cost. Accordingly, this study aimed to determine the efficacy of telerehabilitation in individuals who underwent hand tendon surgery. This randomized controlled study comprised two groups: a home program group and a telerehabilitation group. While the control group continued their routine treatment and home program, the intervention group’s home program was monitored via telerehabilitation three times per week for six weeks. Outcomes were assessed using the Visual Analog Scale (VAS) for pain, a baseline hydraulic hand dynamometer for gross grip strength, a Lafayette hydraulic pinch meter for fine grip strength, the Nine-Hole Peg Test for dexterity, and the Quick Disabilities of the Arm, Shoulder, and Hand (Quick-DASH) questionnaire for functional status. The findings indicated that telerehabilitation had positive effects on activities of daily living and overall functionality.

The hand plays a central role in activities of daily living, sports, and occupational tasks. Consequently, it is frequently overused and is highly susceptible to injury (Pantelić et al., 2022). Industrialization and mechanization have increased the incidence of work-related accidents, which account for a substantial proportion of emergency department visits due to hand injuries (Gwilliam et al., 2025; Mercan et al., 2024; Rodríguez et al., 2024). Common causes include agricultural machinery, as well as cutting, drilling, and pressing tools used in industrial settings. Among these injuries, tendon injuries are the most prevalent (Khurshid et al., 2023; Patel et al., 2025). Such injuries also contribute to an increased workload in emergency departments (Laferté et al., 2020).

Although hand injuries are not typically life-threatening, they may significantly compromise daily functioning and overall quality of life. Notably, patients with hand tendon injuries account for the majority of referrals to hand rehabilitation units (Kablanoğlu et al., 2025). Tendon injuries require long-term, structured rehabilitation programs. Rehabilitation plays a critical role in preventing complications, particularly adhesions, restoring hand function, and facilitating an early return to work (Karaçam and Gultekinler, 2025; Yin and Sun, 2022). Hand tendon injuries often affect working-age adults and can lead to prolonged time away from work, long rehabilitation, and substantial costs for both society and the health system. Moreover, prolonged treatment durations and the risk of complications during or after rehabilitation further exacerbate this burden (Mehrzad et al., 2022). Considering the importance of the hand in daily life and the burdens imposed by treatment, it is necessary to ensure that rehabilitation is planned, effective, and cost-effective (Karaçam and Gultekinler, 2025).

Telerehabilitation has become increasingly widespread in physiotherapy and rehabilitation practice, particularly following the COVID-19 pandemic (Brigo et al., 2022). It is defined as the remote delivery of rehabilitation services through telecommunications technologies (Galea, 2019). Telerehabilitation is an approach that aims to improve access to care by overcoming barriers related to distance, time, and cost (Surya and Someshwar., 2025). Epidemic diseases, particularly the COVID-19 pandemic, have contributed to the widespread adoption of telerehabilitation both globally and in Turkey (Kloze and Wojtal, 2021; Timurtas and Mumcea, 2024). Telerehabilitation has emerged as an effective approach to improving access to care, facilitating healthcare professionals’ work, and reducing the burden on healthcare systems (Nizeyimana et al., 2022). However, the use of telerehabilitation in orthopedic diseases, particularly in the follow-up of hand therapy programs, remains limited. Therefore, this study aimed to investigate the efficacy of telerehabilitation in the follow-up of home programs in individuals who underwent hand tendon surgery using a randomized controlled trial design.

## Materials and Methods

### Study Design

This study was a randomized controlled trial with 18 volunteers who underwent hand surgery at the Physical Therapy and Rehabilitation Department of the Faculty of Medicine, Practice and Research Hospital, Aydın Adnan Menderes University. Approval for the conduct of this study was obtained from the Izmir Democracy University Non-Interventional Clinical Research Ethics Committee, with decision number 2021/05-05 dated 28.04.2021. Individuals who agreed to participate in the study were informed about the study and provided consent. To be included in the study: subjects had sustained an injury to the flexor or extensor tendons of the hand and had undergone corrective surgery; were between the ages of 18 and 65; and had adequate communication skills. Exclusion criteria for the study were: having had prior hand surgery for any reason other than the current injury; not having a smartphone or being unable to participate in the program at home; and lacking reading skills. The sample size for the study was calculated by referencing the study by Hall et al. (2010), which used early passive movement and early active movement data. In the analysis, it was predicted that the minimum number of cases to be included in the groups to achieve 80% power with a type 1 error rate of 0.05 was 9, and our study included 18 cases.

### Assessment

In the first evaluation, information was obtained on individuals’ gender, education level, occupation, dominant hand, hand that has undergone surgery, affected area, and whether they had any previous physiotherapy intervention. Pain, grip strength, and upper extremity function were assessed before and after the study.

#### Pain

Pain was assessed with the Visual Analog Scale (VAS). VAS has been used in social and behavioral sciences to measure various subjective phenomena (Wewers and Lowe, 1990). After explaining to the participant that the starting point represents 0, (i.e., ‘I have no pain’), and the ending point represents 10, (i.e., ‘the worst pain ever’) on the 10 cm horizontal line we drew on the Evaluation Form, they were asked to think about where they felt the pain between these two values and mark it. The marked point was then measured with a ruler, and the result was recorded. Individuals’ pain assessments were obtained at the first and final assessments.

#### Grip Assessment

##### Pinch Strength

A Lafayette brand hydraulic pinchmeter was used for this measurement. It was measured in three ways: tip, key and palmar pinch. In the tip pinch measurement, the pinchmeter was asked to be squeezed with the tip of the thumb and the tip of the index finger. In the key pinch measurement, the volunteer was asked to squeeze the pinchmeter with the middle of the distal phalanx of the thumb and the lateral part of the middle phalanx of the index finger. In palmar grip measurement, the pinchmeter was taught to be squeezed with the thumb and the distal phalanges of the index and middle fingers (Mathiowetz et al., 1984). Measurements were made in the same position as the grip strength, in accordance with the standard. Three measurements were taken for each grip, and the average was recorded in lb. (pounds).

##### Grip Strength

Hand grip strength was assessed with a Baseline hydraulic hand dynamometer. Measurements were made while sitting, with the arm in shoulder adduction, elbow at 90° flexion, and forearm in neutral position. The wrist was placed in 0–30° extension and 0–15° ulnar deviation. The volunteer was asked to grasp the dynamometer firmly and then squeeze it as hard as possible. Measurements were repeated three times, and the average value was recorded in kilograms.

#### Functional Assessment

##### Nine Hole Peg Test

The subjects’ manual dexterity was evaluated using the Nine-Hole Peg Test, which demonstrated validity and reliability. The test consists of nine identical holes and nine identical rods placed at equal intervals (Mathiowetz et al., 1985). The time starts when the person first touches the piece to place it. The person was asked to take the sticks from the box, place them in the holes as quickly as possible, then take them out again and return them to the box. The test was performed with the person seated. The test allowed the platform to be stabilized with the unused hand. Activity duration was recorded in seconds.

##### Quick Disabilities of the Arm, Shoulder and Hand (Quick-DASH)

In our study, the Quick-DASH questionnaire developed by the American Academy of Orthopedic Surgeons was used to evaluate upper extremity functions. Quick-DASH consists of one question and at most one question can be left blank for the score to be calculated (Beaton et al., 2005). The Quick-DASH Turkish adaptation, validity, and reliability were assessed by Doğan et al. (2011).

Study participants were randomized into two groups—the home program group (HPG) and the telerehabilitation group (TRG)—using the online randomization tool Randomizer.org. The total sample size and a 1:1 allocation ratio was entered into the software, and group assignments were determined based on the computer-generated randomization sequence. The same physiotherapist performed the initial and final evaluations. They continued the treatments applied to the cases in both groups and, if any, physical therapy sessions. Personalized home exercises were explained to all patients. After the home programs were explained to patients in the HPG at the first meeting, no intervention was made during the study period. The home programs of the patients in TRG were monitored and followed for 6 weeks through telerehabilitation and online video conferences via the WhatsApp application. The participants attended telerehabilitation sessions on a one-to-one basis. Each session lasted 20 minutes. Home exercise programs were individually tailored based on each patient’s clinical condition. Wand exercises were prescribed to improve the range of motion of the shoulder, elbow, and wrist joints. Elbow and wrist movements were performed with the elbow maintained at a 90° shoulder angle. Tendon gliding and blocking exercises were prescribed for patients with tendon adhesions. The exercises were performed three times per week, with each session lasting approximately 20 minutes and consisting of 10–15 repetitions per exercise. Patients were educated to perform deep friction scar massage to the scar tissue for 5 minutes, 4–5 times per day. In the telerehabilitation group, the application of the technique was additionally monitored during telerehabilitation sessions. Patients’ progress was monitored, and their exercises were either advanced, or new exercises were added based on their individual situation. Patients who received strengthening exercises had their resistance levels adjusted. Patients in both groups were re-evaluated after six weeks.

### Statistical Analysis

All statistical analyses were performed using SPSS 26.0. Statistical information in the tables is presented as sample size (n), sample percentage (%), and mean ± standard deviation. The data obtained in the study were analyzed, and it was determined that they did not conform to the normal distribution. For this reason, non-parametric tests were preferred for the statistical analysis of the data. Therefore, the Mann-Whitney U test was used to determine the significance of the difference between the two groups. The Wilcoxon paired two-sample test was used to evaluate the pre- and post-group data. A p-value of <0.05 was considered significant for all statistical tests.

## Results

Our study began with 39 volunteers at the initial evaluation. Twenty-one subjects were excluded due to re-surgical repair, failure to complete the sessions, or failure to participate in the final evaluation. Ultimately, 18 subjects (HPG 9 and TRG 9) were included in the study.[Fig f1-ijt-18-1-6752]

The distribution of case characteristics recorded in the evaluation form is shown in Table 1.

Participants in the study were randomly divided into two groups: TRG and HPG. When comparing the data obtained from the initial assessment, only the tip grip measurement revealed significant differences between the two groups (p<0.05). There were no significant differences between the groups in other baseline data (p>0.05) (Table 2).

Participants in the TRG and HPG received a final evaluation after 6 weeks. When the pre- and post-treatment data of the groups were compared, positive significance was observed in all TRG pain, grip strength, Nine-Hole Peg Test, and Quick-DASH Questionnaire results (p<0.05). Improvement was observed in the pain, grip strength, Nine-Hole Peg Test, and Quick-DASH data evaluated in the HPG, and the difference was statistically significant (p<0.05). Effect sizes calculated for the statistically significant results across all analyses were consistently large (r > 0.50), suggesting that the intervention exerted a substantial impact on the outcome measures. These results are presented in Table 3.

In the comparison of TRG and HPG between groups, significance was found only in the Quick-DASH data, which evaluates upper-extremity functional status (p=0.047). The effect size of the QuickDASH score was moderate (r=0.46). Although improvements were observed in other data, the difference was not statistically significant (p>0.05) (Table 4).

## Discussion

This study aimed to examine the effectiveness of telerehabilitation in individuals who underwent surgery following hand tendon injuries. Therefore, patients’ home programs were monitored using telerehabilitation. Patients’ pain, grip strength, and functional status were measured to interpret the results. This study found that telerehabilitation was effective in improving upper-extremity function. No changes were observed in other parameters.

Due to its functional role, the hand is highly susceptible to injury. Work, sports, and daily tasks require frequent hand use, which in turn can lead to injuries In addition to the sharp-perforator tools we use daily, industrialization and mechanization also cause serious tendon injuries. It is known that hand injuries are largely caused by occupational accidents (Azm et al., 2024). A study has shown that the majority of people with flexor/extensor tendon injuries in our country are men (Kablanoğlu et al., 2023). Hand tendon injuries often require urgent surgical repair. Evaluation, accurate diagnosis, and referral to the most appropriate treatment are crucial for regaining functionality (Campbell et al., 2020). A successful surgical repair and a well-planned rehabilitation program are crucial for reducing potential complications and accelerating functional return (Svingen et al., 2023). Therefore, post-surgical rehabilitation and exercises must be closely monitored. Telerehabilitation applications enable patients to easily monitor the home rehabilitation process and implement the program regularly (Özden et al., 2020).

Telerehabilitation is an alternative method that provides easy access to treatment for individualsliving in remote areas, those with physical disabilities, transportation difficulties, or problems related to socioeconomic factors (Surya and Someshwar, 2025). It also reduces transportation costs and time spent on both the healthcare professional and the patient (Prada et al., 2024). In our country, the number of patients waiting for treatment in hospital physical therapy and rehabilitation units is quite high. In this regard, we believe that integrating telerehabilitation into health centers will reduce the workload on healthcare personnel and enable them to reach more patients more easily.

Home exercise programs may be less effective when not supervised by a therapist. In a study by Saraee et al. (2017), a platform was developed to monitor patients’ exercises. The purpose of this platform is to monitor patients’ performance and provide feedback on exercise correction if necessary. This platform provides the necessary benefit in monitoring exercises at home (Saraee et al., 2017).

Many studies conducted worldwide indicate an increase in telerehabilitation services, especially following the COVID-19 pandemic (Garcia et al., 2022; Laver et al., 2022; Parraguez et al., 2023; Prvu Bettger and Resnik, 2020; Turolla et al., 2020). The COVID-19 pandemic and the resulting changes in the organization of healthcare facilities have negatively impacted rehabilitation services in many European countries. Telerehabilitation has been considered as a solution to this problem (Kloze andWojtal, 2021). In recent years, the use of telerehabilitation has been shown to lead to clinical improvements equivalent to those of a traditional in-person rehabilitation program (De Cola et al., 2016). Ayhan et al. (2021) reported their experience with telerehabilitation as acceptable and excellent in a study examining current advances in flexor tendon surgery and treatment (Ayhan et al., 2021). However, their experience indicated that good touch is also essential in hand therapy and that telerehabilitation cannot completely replace manual interventions.

Rodríguez Sánchez-Laulhé et al. (2022) investigated the short- and medium-term effectiveness of a phone application (CareHand) in individuals with rheumatoid arthritis in a randomized controlled trial. The study group used an application containing specialized exercise programs and monitoring tools. The control group was given a written home exercise program. At the end of six months, the Quick-DASH score showed significant improvement between the groups, consistent with our study (Rodríguez Sánchez-Laulhé et al., 2022). In a study conducted by Suero-Pineda et al. (2025) comparing a traditional home program with an online application in patients with hand injuries, recovery and resource consumption were investigated. Fewer sessions were needed in the experimental group, the sessions were briefer, and greater improvements were seen in grip strength, pain, and dexterity compared to the control group (Suero-Pineda et al., 2025).

Pech-Argüelles et al. (2023) conducted a randomized controlled trial of 91 patients with distal radius fractures. In that study, the groups received a rehabilitation program for 2 weeks, and the experimental group received a telerehabilitation program with online instructions. At six months, significant differences in functionality were observed within groups, but there was no difference between groups. There was a decrease in pain, and functionality, range of motion, and quality of life increased in both groups (Pech-Argüelles et al., 2023).

Randomized controlled trials on telerehabilitation are quite scarce worldwide (Parraguez et al., 2023). The current original research not only provides practical guidance for healthcare institutions implementing telerehabilitation programs but also informs and supports future research in patient-centered telerehabilitation.

## Clinical Conclusions and Limitations

When planning this study, the primary rationale was the negative impact of the coronavirus pandemic on human life, including social isolation and disruption to daily routines across multiple domains. However, the concurrent increase in COVID-19 case numbers during the data collection period adversely affected participant recruitment and limited access to a sufficient sample size.

## Conclusion

As a result of following home programs with telerehabilitation in patients who underwent hand tendon surgery, it was observed that pain decreased and grip strength, dexterity and functionality increased. It was determined that telerehabilitation was effective to treat upper extremity functionality.

## Figures and Tables

**Figure 1 f1-ijt-18-1-6752:**
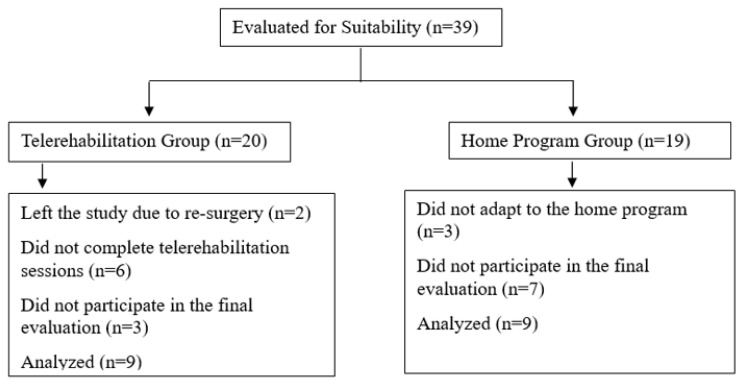
Flowchart of the Study

**Table 1:** Distribution of Case Characteristics

	Variables	TRG (n=9)		HPG (n=9)	

		N	%	N	%
Gender	Female	6	66.7	2	22.2
	Male	3	33.3	7	77.8

Educational Status	Elementary School	1	11.1	6	66.7
	Middle School	2	22.2	1	11.1
	High School	5	55.5	0	0
	University	1	11.1	2	22.2

Occupation	Worker	4	44.4	3	33.3
	Housewife	1	11.1	1	11.1
	Retired	1	11.1	1	11.1
	Self-Employed	2	22.2	0	0
	Student	1	11.1	0	0
	Teacher	0	0	1	11.1
	Driver	0	0	1	11.1
	Barber	0	0	1	11.1
	Farmer	0	0	1	11.1

Dominant Side	Right	8	88.9	7	77.8
	Left	1	11.1	2	22.2

Surgical Side	Right	6	66.7	5	55.5
	Left	3	33.3	4	44.4

Affected Area	Flexor	7	77.8	4	44.4
	Extensor	1	11.1	3	33.3
	Flexor and Extensor	1	11.1	2	22.2

Physiotherapy Intervention	Yes	7	77.8	5	55.5
	No	2	11.1	4	44.4

**Table 2:** Comparison of Data Obtained from the Participants in the Initial Assessment

	Variables	TRG (n=9)	HPG (n=9)	p

		Median (%25–75)	Median (%25–75)	
Pain	VAS (cm)	5 (0–7.75)	2 (0–2.75)	0.489

Grip	Grip Strength (kg)	6 (1.16–11.99)	16.66 (6.99–28.33)	0.051
	Tip Grip (lb)	5.33 (4.58–9.33)	10.33 (8.08–13.16)	**0.019** ^*^
	Key Grip (lb)	10 (4.51–12.16)	12.33 (9.66–18.99)	0.063
	Palmar Grip (lb)	7 (3.83–10.16)	10 (8–18.66)	0.063

Functional Assessment	Nine Hole Peg Test (s)	34 (25.50–54.50)	23 (23–32)	0.136

	Quick-DASH	77.27 (35.90–84.18)	38.63 (15.90–65.90)	0.094

*Note.* VAS: Visual Analog Scale, cm: centimeter, kg: kilogram, lb: libre, s: second, p^*^<0.05, p: Mann-Whitney U

**Table 3:** Comparison of Pre-Treatment and Post-Treatment Changes between Groups

		TRG			HPG		

Variables		Pre-Treatment	Post-Treatment	p/r	Pre-Treatment	Post-Treatment	p/r
			
		Median(%25–75)	Median (%25–75)		Median(%25–75)	Median (%25–75)	
Pain	VAS (cm)	5 (0–7.75)	0 (0–3.75)	**0.043** ^*^ **/0.67**	2 (0–2.75)	0 (0–2)	**0.042** ^*^ **/0.68**

Grip	Grip Strength (kg)	6 (1.16–11.99)	10.66 (4.83–19.33)	**0.008** ^*^ **/0.89**	16.66(6.99–28.33)	17.66 (8.33–31.33)	**0.007** ^*^ **/0.9**
	Tip Grip (lb)	5.33 (4.58–9.33)	10.66 (7.33–15.33)	**0.012** ^*^ **/0.84**	10.33(8.08–13.16)	14 (10.99–16.83)	**0.007** ^*^ **/0.89**
	Key Grip (lb)	10 (4.51–12.16)	10.66 (7.33–12.66)	**0.043** ^*^ **/0.68**	12.33(9.66–18.99)	14.66 (12.33–19.99)	**0.018** ^*^ **/0.79**
	Palmar Grip (lb)	7 (3.83–10.16)	7.66 (5.16–11.33)	**0.008** ^*^ **/0.89**	10 (8–18.66)	12 (9–19.83)	**0.007** ^*^ **/0.9**

Functional Assessment	Nine Hole Peg Test (s)	34(25.50–54.50)	25 (22–38.50)	**0.007** ^*^ **/0.89**	23 (23–32)	22 (20.50–26)	**0.008** ^*^ **/0.89**

	Quick-DASH	77.27(35.90–84.18)	59.09(15.90–78.40)	**0.044** ^*^ **/0.67**	38.63(15.90–65.90)	29.54 (2.27–54.54)	**0.016** ^*^ **/0.89**

*Note.* VAS: Visual Analog Scale, cm: centimeter, kg: kilogram, lb: libre, s: second, p^*^<0.05, p: Wilcoxon Signed Rank Test, r: Effect size

**Table 4:** Comparison of the Data Obtained from the Last Assessment of the Groups

	Variables	TRG	HPG	p/r

		Median (%25–75)	Median (%25–75)	
Pain	VAS (cm)	0 (0–3.75)	0 (0–2)	0.861

Grip	Grip Strength (kg)	10.66 (4.83–19.33)	17.66 (8.33–31.33)	0.297
	Tip Grip (lb)	10.66 (7.33–15.33)	14 (10.99–16.83)	0.113
	Key Grip (lb)	10.66 (7.33–12.66)	14.66 (12.33–19.99)	0.063
	Palmar Grip (lb)	7.66 (5.16–11.33)	12 (9–19.83)	0.094

Functional Assessment	Nine Hole Peg Test (s)	34 (25.50–54.50)	22 (20.50–26)	0.222

	Quick-DASH	59.09 (15.90–78.40)	29.54 (2.27–54.54)	**0.047** ^*^ **/0.46**

*Note.* VAS: Visual Analog Scale, cm: centimeter, kg: kilogram, lb: libre, s: second, p^*^<0.05, p: Mann-Whitney U
